# Using the Nursing Culture Assessment Tool (NCAT) in Long-Term Care: An Update on Psychometrics and Scoring Standardization

**DOI:** 10.3390/healthcare3030637

**Published:** 2015-07-29

**Authors:** Susan Kennerly, Eric D. Heggestad, Haley Myers, Tracey L. Yap

**Affiliations:** 1 School of Nursing, University of North Carolina at Charlotte, Charlotte, NC 28223, USA; 2 Department of Psychology and Organizational Science, University of North Carolina at Charlotte, Charlotte, NC 28223, USA; E-Mail: edhegges@uncc.edu; 3 Department of Organizational Science, University of North Carolina at Charlotte, Charlotte, NC 28223, USA; E-Mail: hmyers11@uncc.edu; 4 School of Nursing, Duke University, Durham, NC 27710, USA; E-Mail: tracey.yap@duke.edu; 5 Center for the Study of Aging and Human Development, Duke University, Durham, NC 27710, USA

**Keywords:** nursing culture, assessment, occupational subculture, long-term care

## Abstract

An effective workforce performing within the context of a positive cultural environment is central to a healthcare organization’s ability to achieve quality outcomes. The Nursing Culture Assessment Tool (NCAT) provides nurses with a valid and reliable tool that captures the general aspects of nursing culture. This study extends earlier work confirming the tool’s construct validity and dimensionality by standardizing the scoring approach and establishing norm-referenced scoring. Scoring standardization provides a reliable point of comparison for NCAT users. NCAT assessments support nursing’s ability to evaluate nursing culture, use results to shape the culture into one that supports change, and advance nursing’s best practices and care outcomes. Registered nurses, licensed practical nurses, and certified nursing assistants from 54 long-term care facilities in Kentucky, Nevada, North Carolina, and Oregon were surveyed. Confirmatory factor analysis yielded six first order factors forming the NCAT’s subscales (Expectations, Behaviors, Teamwork, Communication, Satisfaction, Commitment) (Comparative Fit Index 0.93) and a second order factor—The Total Culture Score. Aggregated facility level comparisons of observed group variance with expected random variance using r_wg(J)_ statistics is presented. Normative scores and cumulative rank percentages and how the NCAT can be used in implementing planned change are provided.

## 1. Introduction

Organizational culture is believed to affect workplace safety, efficiency, and effectiveness; in long-term care (LTC) settings the high rate of staff turnover further complicates instability of the occupational subculture of nursing (the primary oversight of resident safety and health) [1]. The nursing’s occupational subculture (hereafter referred to as nursing culture), includes licensed nurses (registered nurses [RN] and licensed practical nurses [LPN]), as well as certified nursing assistants (CNAs), all of whom must function as a nursing team. The need within healthcare to promote the retention of and successful performance of the nursing workforce has long been recognized [2,3,4]. Turnover affects staffing decisions and adds to organizational costs associated with reduced worker efficiency and productivity because of the instability in the workforce that results, especially when high performing staff leave and must be replaced [5,6,7]. High turnover rates for licensed and non-licensed nursing staff can be found in all types of healthcare settings. In fact, difficulty recruiting and retaining CNAs occurs in 45 states [8,9]. In LTC settings, turnover rates are as high as 80%–85% for CNAs and typically occur in the first 90 days of employment. Staffing levels and workload in LTC are reported as potential contributors to turnover [10]. CNAs’ integration of social structural processes is important since CNAs are the frontline workers, doing 90% of direct care [11]. Literature shows stabilized, lower turnover rates may be linked to effectual cultures [12]. Thus, creation of a positive workplace culture is crucial to worker and LTC resident safety and effective and appropriate care outcomes in high turnover LTC organizations [13,14]. Relationships among nursing staff are derived from the workplace cultural environment, which emerges from the values, attitudes, and beliefs held by staff, and make a critical contribution to the organization’s ability to achieve quality care outcomes.

The creation of a positive workplace culture is a necessary foundation for workers to be able to see and experience the benefits of cultural elements such as accountability, autonomy, safety, and teamwork, and to become committed to the organization and experience satisfaction with the job [15,16]. Similarly, perceived organizational support [17] and affective organizational commitment [18] have also been shown to have links to organizational culture and worker satisfaction and job performance. Organizations that wish to bring about and/or sustain change in quality care and workplace practices face the challenge of creating a culture of accountability and safety with regard to those practices. Effective cultural change begins with establishing clear, non-threatening communication channels throughout the organization and requires strong leadership and workforce stability. The first step in enabling nurse leaders to shape a positive nursing workplace culture is a comprehensive assessment of the occupational subculture of the nursing workforce.

The Nursing Culture Assessment Tool (NCAT) was developed to provide administrators, clinicians, and researchers with a reliable and valid means of assessing the occupational subculture of nursing. We applied a rigorous mixed methods approach to developing and refining the instrument [19,20,21]. This study takes the NCAT to its next level of development by reconfirming the tool's construct validity and dimensionality in a larger sample and by standardizing the scoring approach through norm-referenced scoring. NCAT users can now compare LTC clinical unit or facility assessment results to standardized scores providing a common perspective from which to use assessment results to evaluate and shape the culture into one that could better support project success when implementing planned change, thereby advancing nursing’s best practices and care outcomes. We report on the results scoring standardization process and offer insights about how the NCAT can be used by LTC leaders to conduct a baseline assessment of the nursing culture and design and implement intervention strategies aimed at bringing about a positive nursing culture prior to introducing change in the workplace.

## 2. Experimental Section

### 2.1. Design

A cross-sectional design and a survey approach was used to collect demographics and survey data. Confirmatory factor analysis of the NCAT’s items and determination of percentile scores were conducted to establish a norm-referenced approach to NCAT scoring and interpretation. This study builds on the already established face and content validity of the 19-item NCAT by analyzing the performance of the measure when completed by a representative sample of the RNs, LPNs, and CNAs working in LTC facilities.

### 2.2. Setting and Participants

LTC facilities (*n* = 54) from a convenience sample in urban and rural areas of North Carolina, Kentucky, Nevada, and Oregon participated. Researchers contacted the facility director of nursing for permission to include the facility in the study. All willing and consented part-time or full-time nursing staff (RN, LPN, and CNA) with the ability to read and understand English and who had worked at the respective facility for 3 or more months were eligible to participate in the study. Staff ages (≥18-years-old) and staff of all races, ethnicities, physical ability, and gender were eligible for inclusion. The norming process was implemented through administration of the NCAT to at least 5 nursing staff (RNs, LPNs, and CNAs) at each facility. Before agreeing to participate in this study, participants were informed of the purpose, procedures, risks, and potential benefits of the study through a statement of informed consent. The University of North Carolina Charlotte Institutional Review Board approved the study prior to data collection.

### 2.3. Power Analysis

Based on a minimum sample of 300 nurse and CNA survey respondents, there was 80% power to demonstrate that the Cronbach’s alpha (α) statistic calculated for the NCAT and its subscales or a correlation of the NCAT or its subscales with the IES-R is significantly different from zero if any of these statistics exceed 0.14 and 90% power for statistics that exceed 0.17. The precision of an α statistic, as measured by its 95% confidence interval, that is in the 0.70 to 0.90 range is approximately ±0.04 to ±0.01, indicating that 95% of the time we would not overestimate a (population value of) Cronbach’s α of less than 0.66 as greater than 0.7 with a sample size of 300.

### 2.4. Measurement—Survey Instrument

The survey instrument was composed of demographic and NCAT items. An investigator developed demographic data collection tool was used to ascertain, age, gender, type of licensed/certified job role, years in current position, and LTC facility name from each participant. The NCAT [19,20] is a 19 item questionnaire composed of declarative statements about nursing and culture in the work setting. Respondents were instructed to rate each item from his/her own perceptions about the healthcare work environment in which currently employed. Each respondent was asked to assign a rating of each of the items according to an ordinal scale, including Strongly Disagree (1); Disagree (2); Agree (3); and Strongly Agree (4). Six core dimensions were hypothesized as being represented within the 19 items. These six subscales were proposed as Expectations (Items 1–3), Behavior (Items 4–6), Teamwork (Items 7–10), Communication (Items 11–13), Satisfaction (Items 14, 15), and Professional Commitment (Items 16–19).

### 2.5. Data Collection/Methods/Procedures

LTC facilities were identified from the Centers for Medicare and Medicaid list of Medicare certified LTC facilities. This list is publically available from www.medicare.gov/nursinghomecompare data regarding the facility size, 5 star rating, and quality measures, such as staff care hours per resident per day were collected. Each facility’s director of nursing was contacted by phone and followed up with an email including a description of the study and procedures and a consent form for signature that formally requested the facility’s participation. The study was explained, sample study materials provided, and permission requested to distribute study materials to nursing staff. Once the facility administrator had agreed to participation, a time was determined for administering the survey to staff and a staff recruitment flier was posted at the facility. Data were collected via either printed survey or electronically from study participants using Qualtrics online software (Qualtrics, Provo, UT, USA, 2014). All data were combined into a single Excel database for statistical analyses. Use of a subject identification number permitted grouping of staff participants by the respective facility.

### 2.6. Data Analysis

Data were transferred into SPSS or M-Plus for data analysis. NCAT scores were calculated for each item, subscale, and total score by assigning the numerical value to the answer marked for each item (*strongly disagree* = 1, *disagree* = 2, *agree* = 3, *strongly agree* = 4). Reverse scoring is not indicated for any item, so subscale and total score calculation was achieved by a simple summing process. Statistical analysis included analysis of demographic data, psychometric evaluation of the NCAT, and norming procedures to obtain percentile ranks. Multigroup Equivalence/Invariance analysis (ME/I) was used to evaluate whether the factor structure of the culture measure was the same in the data collection groups. The psychometric evaluation of the NCAT included an examination of Cronbach’s Alpha reliability and factor structure using Confirmatory Factor Analyses (CFA). The chi-square statistics comparative fit index (CFI), the root mean-square error of approximation (RMSEA) were used as indicators of first and second order factors. Structural analyses of item functioning, including item cross-loadings and inter-item correlations were also examined. Aggregation from individual level responses to facility level scores was supported by examination of the r_wg(J)_ index of agreement.

## 3. Results and Discussion

Empirical construct validity and dimensionality of the NCAT tool were confirmed and norm-referenced scoring was established with a total of 1025 predominately female nursing staff (204 RNs, 243 LPNs, and 578 CNAs) from 54 LTC facilities participating in the study. The mean LTC facility size was 117 Medicare and Medicaid certified beds with a mean of 99 total residents and mean 5 Star rating of 3 (range = 1 to 5). Mean nursing hours per resident day for RN, LPN, and CNA care were 46, 49, and 139, respectively.

### 3.1. NCAT’s Dimensionality Supported

We examined the fit of the data to a hypothesized model that included items as indicators of six first order factors (the subscales) and a second-order factor representing Overall Culture. The model was found to fit the data well (CFI = 0.932; RMSEA = 0.078; SRMR = 0.048) (Table 1). The standardized loadings for the first-order factor ranged from 0.631 to 0.912 and the loadings for the second-order factor ranged from 0.398 to 0.965 (Table 2). Cronbach’s alpha coefficients of items in each subscale and in the overall scale ranged from 0.71 to 0.94. The nursing staff’s mean NCAT Total score was 59.21 with the full range of NAT scores from 19 to 76 represented. Mean subscale scores were Expectations 9.32 (3 items), Behavior 9.31 (3 items), Communication 9.22 (3 items), Teamwork 11.22 (4 items), Satisfaction 5.75 (2 items), and Commitment 14.37 (4 items). Standardized loadings for each item ranged from 0.631 to 0.912 (Table 2).

**Table 1:** The Nursing Culture Assessment Tool’s (NCAT) Comparative Fit Index, Standardized Root Mean Square, and Reliability Estimates.

Statistical Procedure (*n* = 1025)	Finding
Comparative Fit Index (CFI)	0.932
Standardized Root Mean Square (SRMR)	0.048
Cronbach’s Alpha Reliability Coefficients:	
NCAT Total Score	0.94
NCAT Subscale Scores:	
• Expectations	0.85
• Behaviors	0.71
• Teamwork	0.91
• Communication	0.79
• Satisfaction	0.82
• Professional Commitment	0.91

Pearson product-moment correlation coefficients between each pair of NCAT subscales was at a medium (≥0.05) to high (≥0.8) level ranging from 0.60 to 0.71 for all subscales except for Commitment (*r* = 0.30 to 0.335; low <0.5), which focuses on perceptions of self and professional commitment rather than relationships with other staff in the facility. The correlation between Commitment and the NCAT Total Score is 0.553.

**Table 2:** Standardized Factor Loadings and Standard Error (*n* = 1025).

Subscale	Item #	Factor Loading	Standard Error
Expectations	1	0.869	0.011
	2	0.873	0.011
	3	0.727	0.018
Behaviors	4	0.631	0.021
	5	0.746	0.018
	6	0.676	0.020
Teamwork	7	0.780	0.014
	8	0.859	0.010
	9	0.863	0.010
	10	0.861	0.010
Communication	11	0.740	0.017
	12	0.685	0.019
	13	0.809	0.014
Satisfaction	14	0.804	0.014
	15	0.869	0.013
Commitment	16	0.855	0.010
	17	0.772	0.014
	18	0.912	0.008
	19	0.852	0.010
Overall Culture Scale	Expectations	0.820	0.016
	Behaviors	0.962	0.015
	Teamwork	0.831	0.014
	Communication	0.965	0.011
	Satisfaction	0.891	0.013
	Commitment	0.398	0.029

### 3.2. NCAT’s Normative Scores

Normative results are based on the aggregation of data to the facility level (*n* = 54). Using r_wg(J)_ statistics, aggregated facility level comparisons showed acceptable ≥0.50 values for each scale supporting the inclusions of all 54 facilities in the aggregation. Normative scores for the overall Nursing Culture Total Score (summative for all 6 subscales) are presented in Table 3. The tabled values indicate the percentage of 54 LTC facilities with scores < that level. For example, at a score of 69, 98.2% of sites had scores at or below 69. Similarly, Cumulative Rank Percentages are listed in Table 4 for each subscale. A Total subscale score of 7 for Expectations shows that 1.8% of all sites had scores of <7 for Expectations. The percentages in Table 4 and Table 5 can be applied by NCAT users to compare how their respective NCAT results compare to those of other LTC sites.

**Table 3:** Cumulative Rank Percentage of Facility Scores on Nursing Culture Total Score.

Nursing Culture Total Score					
Total Score	Cumulative % Rank	Total Score	Cumulative % Rank	Total Score	Cumulative % Rank
76	100	57	30.9	38	0
75	100	56	25.5	37	0
74	100	55	12.7	36	0
73	100	54	9.1	35	0
72	100	53	3.6	34	0
71	100	52	3.6	33	0
70	100	51	3.6	32	0
69	98.2	50	1.8	31	0
68	96.4	49	0	30	0
67	96.4	48	0	29	0
66	92.7	47	0	28	0
65	90.9	46	0	27	0
64	89.1	45	0	26	0
63	87.3	44	0	25	0
62	81.8	43	0	24	0
61	72.7	42	0	23	0
60	58.2	41	0	22	0
59	43.6	40	0	21	0
58	38.2	39	0	20	0
				19	0

**Table 4:** Cumulative Rank Percentage for Each of the NCAT’s Six Subscale Total Scores.

Cumulative Rank Percentage						
Total Score	Expectations	Behaviors	Teamwork	Communication	Satisfaction	Professional Commitment
16			100			100
15			100			92.7
14			100			52.7
13			92.7			9.1
12	100	100	87.3	100		1.8
11	98.2	100	58.2	98.2		0
10	94.5	92.7	25.5	90.9		0
9	50.9	58.2	3.6	56.4		0
8	12.7	10.9	0	10.9	100	0
7	1.8	0	0	0	98.2	0
6	0	0	0	0	92.7	0
5	0	0	0	0	27.3	0
4	0	0	0	0	1.8	0
3	0	0		0	1.8	
2					1.8	

### 3.3. Using the NCAT to Guide Change

Positive work and care environments provide a safe, respectful, and civil atmosphere in which to implement job responsibilities. A “negative healthcare work environment” has been consistently cited by licensed nurses as being among the top reasons difficulty is encountered in recruiting and retaining licensed nurses in the workforce [4]. Similarly, difficulty recruiting and retaining CNAs occurs in most states [11]. Literature shows stabilized, lower turnover rates may be linked to effectual cultures [7]. In long-term care organizations, culture is thought to be a strong factor in determining the organization’s ability to achieve Quality Measures and Quality Indicators (QI), such as prevention of pressure ulcers and falls. In fact, one QI is staff turnover rate, which is believed to be a key disruptor of culture and a strong influence on quality and environment. Disenchanted staff will often generate sub-cultures that deepen dissatisfaction and contribute to high turnover rates [5]. In contrast, a positive culture leads to teamwork, safe behavior, satisfaction, commitment, and good communication [22]; therefore, leading to decreased stress, thereby reducing the impact risks both short and long-term on the ability of licensed nurses and CNAs to safely do their jobs. The NCAT is a valid and reliable nursing culture assessment tool that nurse leaders can use in first assessing the nursing work culture. This study’s results specific to scoring and score interpretation offer a common point for comparison and could lead to changes in workplace environments that will be effective in improving care outcomes.

The NCAT’s normative subscale scores and cumulative rank percentages can be used in interpreting baseline nursing culture assessment and periodic reassessment results when planning change and implementing improvements in care practices. Table 5 presents seven core steps that we believe are foundational for NCAT users to include when interpreting NCAT results and then applying them to facilitate a transition in their own facility’s nursing culture to a more positive one. Administering the NCAT (Items available in earlier publication [20]) to all nursing staff and scoring the NCAT as described above will provide a baseline picture of staff perceptions of their workplace nursing culture. Next, compare the facility performance and that of individual clinical units as indicated to the normative NCAT total score in Table 3 and note the Cumulative Rank Percentage corresponding to your facility/unit mean score. If your mean score is below 65, only 10.9% of facilities scored higher. You may find it useful to further explore your performance and discover which subscale(s) contributed to the lower scores at your facility. Compare facility/unit mean subscale scores to the Cumulative Rank Percentage given in Table 4 for each subscale in order to further identify potential areas in which improvement in staff relationships might be indicated. For example, Teamwork in the change exemplar has a cumulative percentage of only 58.2, which could be considered less than desirable if planning to launch a QI improvement project that requires a focused team effort. Targeting this nursing culture dimension for improvement can be facilitated by examining staff ratings for Teamwork subscale items. For example, team building activities might best be focused on clarifying group concerns if among staff performance on the Teamwork subscale items you find that the lowest rated item by staff is “Staff show respect for one another”. Strategies for clarifying expectations regarding respect and defining group activities to help staff share in achievement of mutually set goals will be essential components of an improvement plan. After an appropriate period of time working on improvement strategies, such as 3 to 6 months, re-administer the NCAT and evaluate the impact of strategies on nursing culture. Benchmark your facility’s/unit’s progress and determine whether your next step is to revise your improvement plan and/or move to introduce your QI planned change project to advance nursing’s best practices and care outcomes.

**Table 5:** Steps for Guiding Implementation of a Planned Change Exemplar.

Steps	Planned Change Exemplar		
Administer and score the NCAT to establish baseline	All RNs, LPNs, CNAs at your Facility		
Compare unit/facility performance to Normative NCAT Total Score	Facility Mean Score = 60 (Cumulative Rank 58.2%)		
Compare facility mean subscale score to Cumulative Rank Percentage for respective subscale.	Subscale	Mean	Cum %
	Behaviors	9.8	92.7%
	Expectations	9.44	50.9%
	Teamwork	10.78	58.2%
	Communication	9.7	90.9%
	Satisfaction	6.33	92.7%
	Prof Commitment	14.39	52.7%
Identify dimension(s) for targeted improvement	Clarify Expectations; strengthen Teamwork		
Engage in improvement planning	Develop and implement plan: Clarify Expectations; Group activities to strengthen Teamwork		
Re-administer NCAT	Prior to introducing change and 3 to 6 month intervals as indicated		
Evaluate progress	Revise improvement plan accordingly		

## 4. Conclusions

The NCAT item structure yields a reliable and valid instrument that is representative of the construct of nursing culture. With this instrumentation available, LTC leaders are able to assess the nursing culture, thereby enabling them to design and implement intervention strategies aimed at bringing about a positive nursing culture. Results specific to scoring and score interpretation from this research will offer a common point for comparison and could lead to changes in workplace environments that will be effective in improving care outcomes. Furthermore, by better understanding the workplace culture and its effect on licensed nurses and CNAs, it will be possible to design intervention strategies to reduce psychosocial stressors, thus reducing turnover and its latent impact on workload, worker safety, and healthcare practices.
